# Supplementary material for: Advancing our understanding of genetic risk factors and potential personalized strategies for pelvic organ prolapse
Source: Nat Commun. 2022 Jun 23;13:3584. doi: 10.1038/s41467-022-31188-5 (PMC9226158; doi:10.1038/s41467-022-31188-5)
Supplement: Supplementary file 1 — Supplementary Information [file 41467_2022_31188_MOESM1_ESM.pdf]

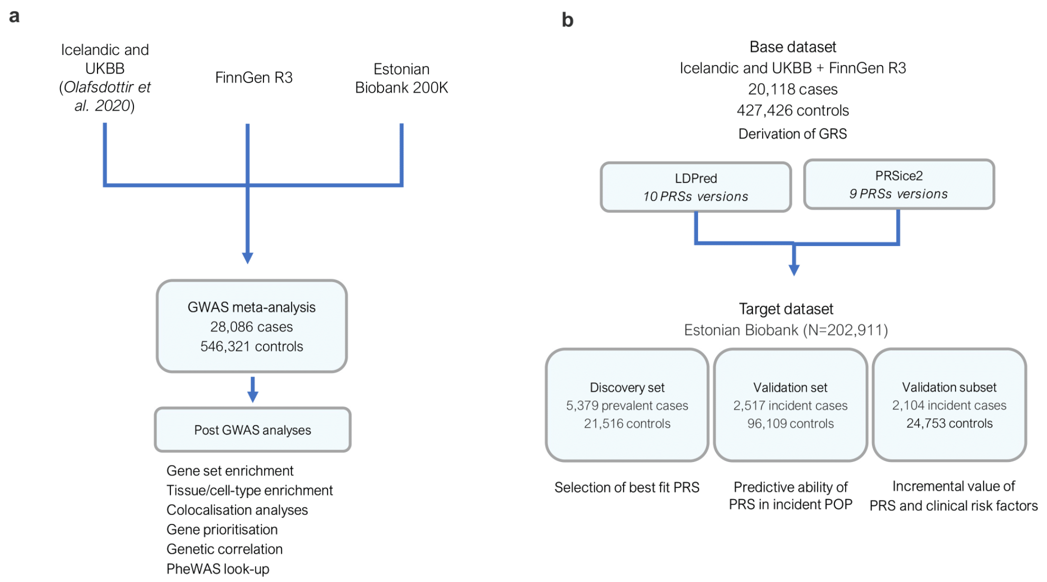

Supplementary Figure 1. Study design. A) Three European ancestry case-control studies were meta-analysed containing a total of 28,086 women with POP and 548,321 controls. Post-GWAS analyses guided the nomination of candidate genes and established genetic and phenotypic links. B) Derivation of PRS from base dataset and PRS construction and validation using Estonian Biobank as target dataset. Target dataset was further split into discovery and validation set and subset. UKBB: UK Biobank, GWAS; genome-wide association study, PRS: polygenic risk score.

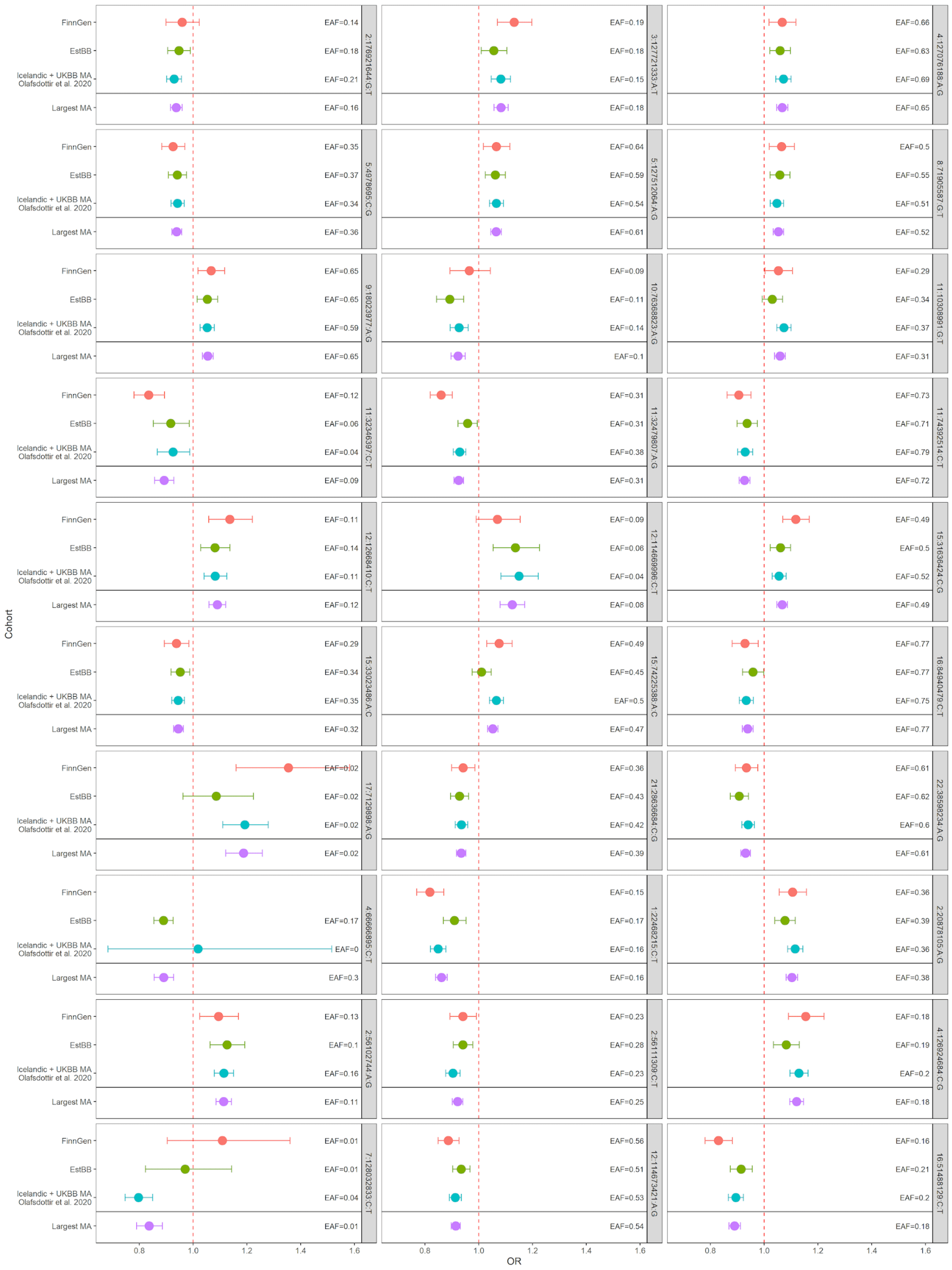

Supplementary Figure 2. Forest plot of effect estimates for the 30 lead variants associated with pelvic organ prolapse accross datasets meta-analysed. Data are presented as mean values +/- SD (95%CI). The odds ratios (dots) and 95% confidence intervals (error bars) are shown for the three studies meta-analysed (FinnGen (red dots, *n*=5,518 cases and 43,366 controls), EstBB (green dots, *n*=7,896 cases and 188,865 contols), Icelandic+UKBB (blue dots, *n*=15,010 cases and 340,734 controls) and the presented largest meta-analysis (Largest MA, purple dots, *n*=28,086 cases and 546,291 controls). Odds ratio represent the effect sizes of each genetic variant association to phenotype, assessing the same risk allele across cohorts (Effect allele as stated in Table 1).

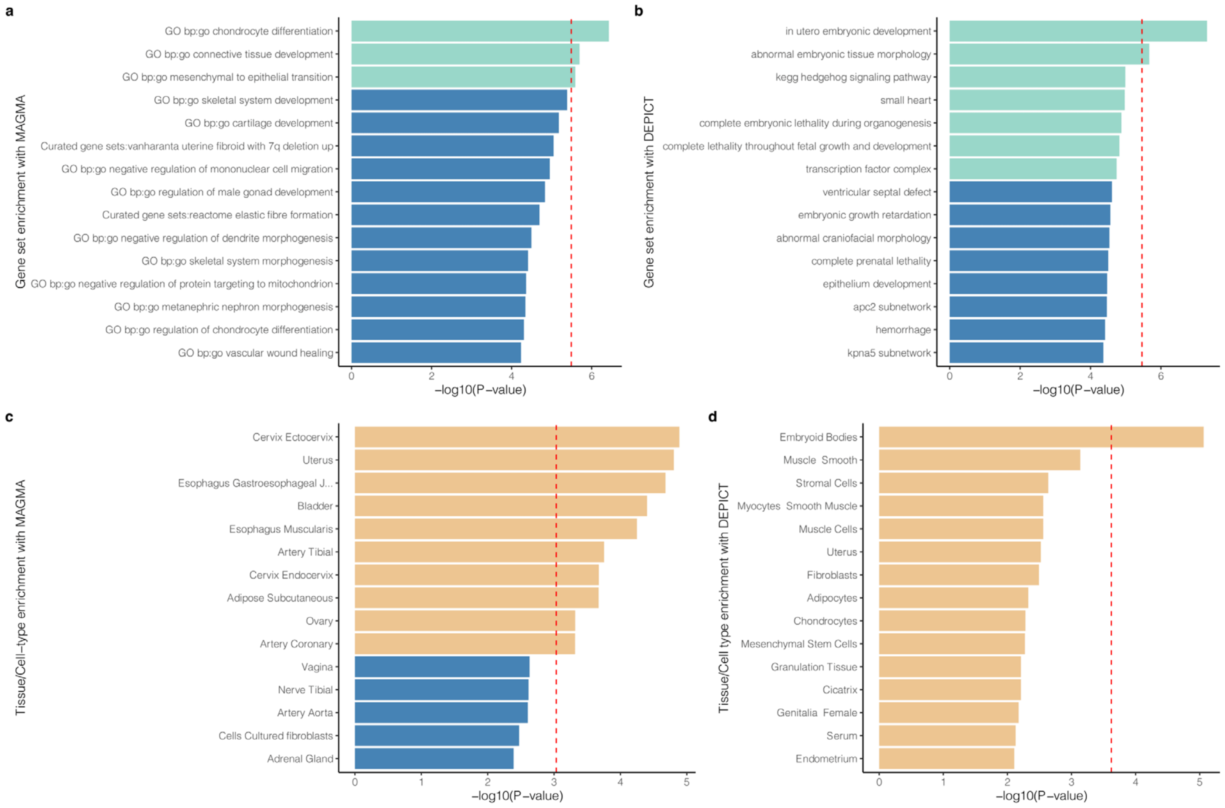

Supplementary Figure 3. Red dashed lines indicate threshold for significance after adjustment for multiple comparisons as detailed below. **a) Gene set enrichment analyses with MAGMA.** Green bars and the red dashed line indicate Bonferroni threshold, set to p=0.05/15485=3.22 × 10^{-6}. **b) Gene set enrichment analyses with DEPICT.** FDR corrected results (p<0.00001) are shown in green bars and the red dashed line indicates Bonferroni threshold set to p=0.05/14461=3.45 × 10^{-6} **c) Tissue enrichment analyses with MAGMA.** Orange bars and the red dashed line indicate Bonferroni threshold, set to p=0.05/54=0.0009**.** **d) Tissue/cell-type enrichment analyses with DEPICT.** FDR corrected results (p<0.00001) are shown in orange and the red dashed line indicates Bonferroni threshold set as p=0.05/209=0.0002

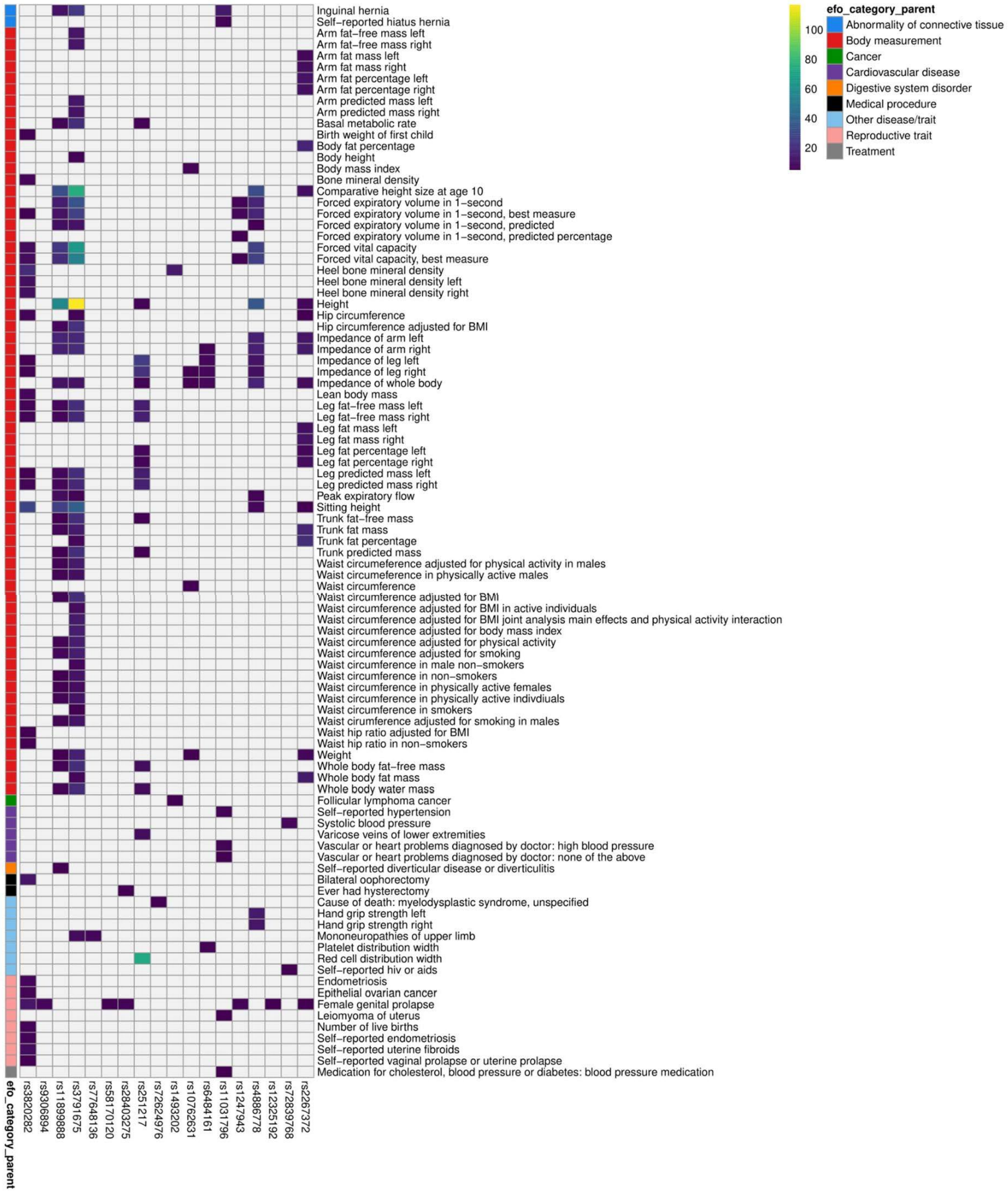

Supplementary Figure 4. Phenoscanner look-up results. Heatmap showing the pheWAS associations with the GWAS lead variants. Traits are grouped based on the experimental factor ontology (EFO) terms, and EFO terms with few traits grouped into one group, “Other disease/trait”. Tiles are colored by the -log10(GWAS P-value) or grey, if there was no suggestive signal obtained for the given variant and trait (GWAS P-value > 10-5).

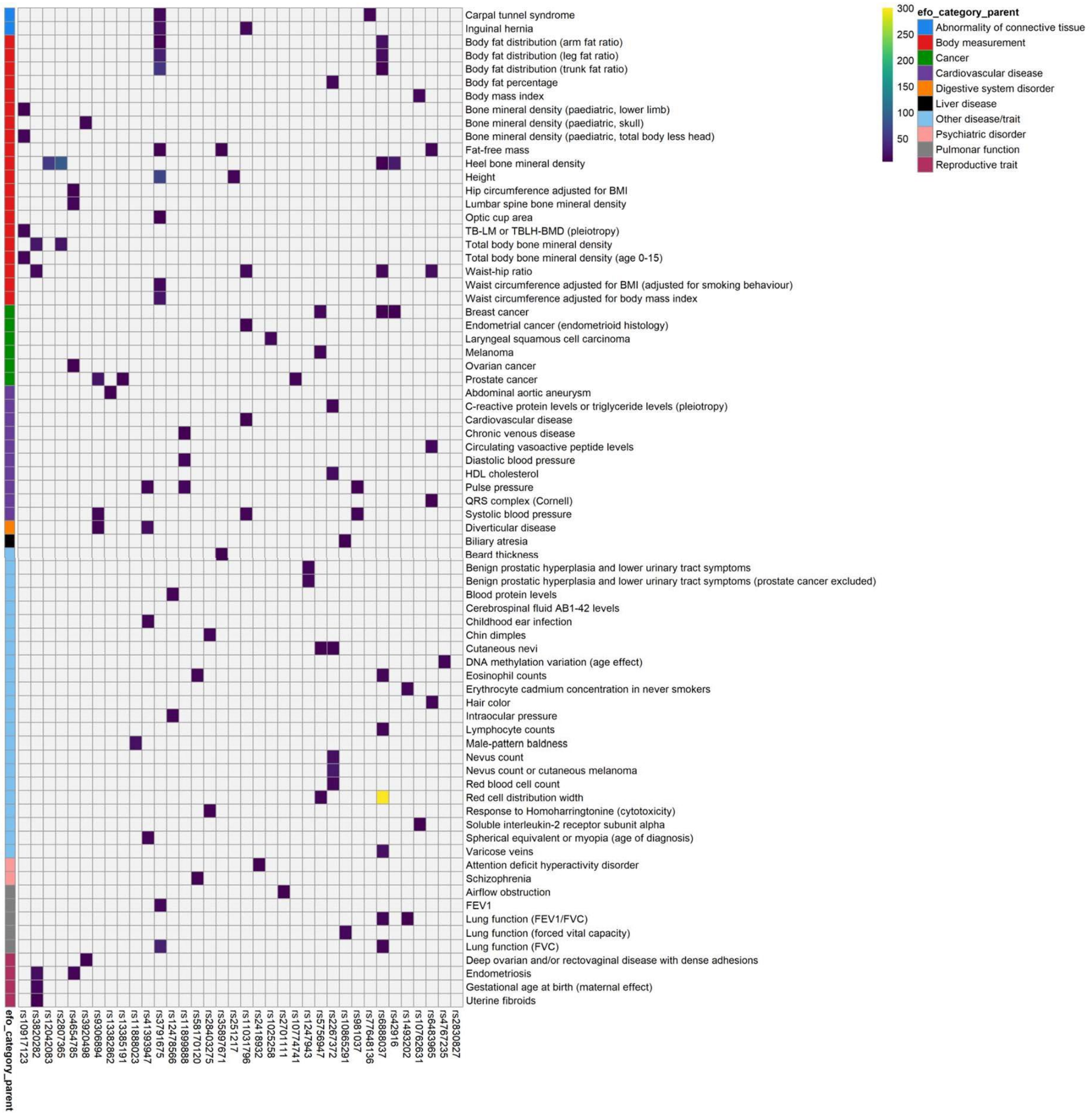

Supplementary Figure 5. GWAS catalog results. Heatmap showing the pheWAS associations with the GWAS lead variants and variants in high LD (r2>0.6) with these. Traits are grouped based on the experimental factor ontology (EFO) terms, and EFO terms with few traits grouped into one group, "Other disease/trait". Tiles are colored by the -log10(GWAS P-value) or grey, if there was no suggestive signal obtained for the given variant and trait (GWAS P-value > 10-5).

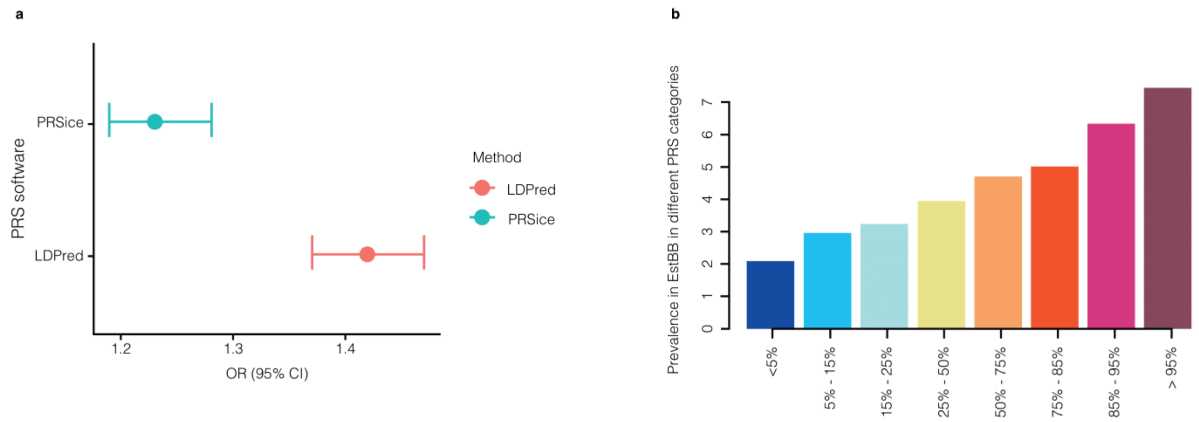

Supplementary Figure 6. A) Comparison of best-fit polygenic risk score model generated by PRSice (blue dot) and LDpred. (red dot). Data are presented as mean values +/- SD (95%CI). OR and 95% CI were obtained from running a logistic regression assessing the discriminative ability of the polygenic risk score (PRS) towards case-control association in discovery set of Estonian Biobank (*n*=5,379 prevalent cases and 21,516 controls). Analyses were adjusted by age, age squared, batch effects and first 10 principal components. B) POP prevalence (%) between different PRS distributions in Estonian Biobank.
