# Supplementary material for: Advancing our understanding of genetic risk factors and potential personalized strategies for pelvic organ prolapse
Source: Nat Commun. 2022 Jun 23;13:3584. doi: 10.1038/s41467-022-31188-5 (PMC9226158; doi:10.1038/s41467-022-31188-5)
Supplement: Supplementary file 3 — Description of Additional Supplementary Files [file 41467_2022_31188_MOESM3_ESM.pdf]

Description of Additional Supplementary Files

File Name: **Supplementary Data 1**
Description: Results for the genome-wide significant index variants in the 26 loci associated with POP identified in the GWAS meta-analysis of 28,086 women with POP and 546,291 female controls. Positions are according to build GRCh37. Bonferroni correction was used to account for multiple testing in Q-Cochran heterogeneity test, with a threshold set to p= 0.05/30.

File Name: **Supplementary Data 2**
Description: Association results of the three studies meta-analysed. Results for the genome-wide significant index variants in the 26 loci associated with POP identified in the GWAS meta-analysis of 28,086 women with POP and 546,291 female controls are shown.

File Name: **Supplementary Data 3**
Description: Results for colocalization analyses, including signals that showed a posterior probability for a shared causal variant (PP4) > 0.8).

File Name: **Supplementary Data 4**
Description: Gene set results with MAGMA. Bonferroni correction was used to account for multiple testing, with a threshold set to p= 0.05/15,488.

File Name: **Supplementary Data 5**
Description: Gene set significant results with DEPICT (FDR was used for multiple testing adjustment and set as FDR < 0.00001)

File Name: **Supplementary Data 6**
Description: Tissue enrichment set with MAGMA. Bonferroni corrected was used to account for multiple testing, with a threshold set to p= 0.05 / 54.

File Name: **Supplementary Data 7**
Description: Tissue/cell-type enrichment significant results with DEPICT (FDR was used for multiple testing adjustment and set as FDR < 0.00001)

File Name: **Supplementary Data 8**
Description: Genetic correlations of POP with 516 traits. Bonferroni correction was used to correct for multiple testing, with a threshold set to p=0.05/561

File Name: **Supplementary Data 9**
Description: Phenoscanner look-up.

File Name: **Supplementary Data 10**
Description: GWAS catalog look-up.

File Name: **Supplementary Data 11**
Description: Discriminative ability of 19 PRS models. A logistic regression was used to assess the discriminative ability towards case-control association. Analyses were adjusted by age, age squared, batch effects and first 10 principal components.

File Name: **Supplementary Data 12**
Description: Summary statistics between cases and controls through different groups defined are given.

File Name: **Supplementary Data 13**
Description: Association between polygenic risk score and different age categories in the validation set of Estonian Biobank.

File Name: **Supplementary Data 14**
Description: Estonian Biobank cohort characteristics for validation set and subset.

File Name: **Supplementary Data 15**
Description: Harrell C-statistic in the validation subset for PRS and clinical risk factors alone or in combination.
