# Supplementary material for: Advancing our understanding of genetic risk factors and potential personalized strategies for pelvic organ prolapse
Source: Nat Commun. 2022 Jun 23;13:3584. doi: 10.1038/s41467-022-31188-5 (PMC9226158; doi:10.1038/s41467-022-31188-5)
Supplement: Supplementary file 5 — Reporting Summary [file 41467_2022_31188_MOESM5_ESM.pdf]

nature portfolio

Corresponding author(s): Natàlia Pujol Gualdo

Last updated by author(s): May 3, 2022

# Reporting Summary

Nature Portfolio wishes to improve the reproducibility of the work that we publish. This form provides structure for consistency and transparency in reporting. For further information on Nature Portfolio policies, see our Editorial Policies and the Editorial Policy Checklist.

## Statistics

For all statistical analyses, confirm that the following items are present in the figure legend, table legend, main text, or Methods section.

| n/a | Confirmed | |
|---|---|---|
| ☐ | ☒ | The exact sample size (*n*) for each experimental group/condition, given as a discrete number and unit of measurement |
| ☒ | ☐ | A statement on whether measurements were taken from distinct samples or whether the same sample was measured repeatedly |
| ☐ | ☒ | The statistical test(s) used AND whether they are one- or two-sided<br>*Only common tests should be described solely by name; describe more complex techniques in the Methods section.* |
| ☐ | ☒ | A description of all covariates tested |
| ☐ | ☒ | A description of any assumptions or corrections, such as tests of normality and adjustment for multiple comparisons |
| ☐ | ☒ | A full description of the statistical parameters including central tendency (e.g. means) or other basic estimates (e.g. regression coefficient) AND variation (e.g. standard deviation) or associated estimates of uncertainty (e.g. confidence intervals) |
| ☐ | ☒ | For null hypothesis testing, the test statistic (e.g. *F*, *t*, *r*) with confidence intervals, effect sizes, degrees of freedom and *P* value noted<br>*Give P values as exact values whenever suitable.* |
| ☐ | ☒ | For Bayesian analysis, information on the choice of priors and Markov chain Monte Carlo settings |
| ☒ | ☐ | For hierarchical and complex designs, identification of the appropriate level for tests and full reporting of outcomes |
| ☐ | ☒ | Estimates of effect sizes (e.g. Cohen's *d*, Pearson's *r*), indicating how they were calculated |

*Our web collection on statistics for biologists contains articles on many of the points above.*

## Software and code

Policy information about availability of computer code

**Data collection**

Information on how genotype and phenotype data was collected is available in Supplementary Information. We used UCSC Liftover software (open-source) to liftover data from different builds

**Data analysis**

The following standard software packages were used for the analyses described in the manuscript:
GenomeStudio (v2.0.4), Eagle (v2.3), and Beagle (v28Sep18.793) were used as part of the standard genotyping and imputation pipeline
SAIGE (v0.38) was used for genome-wide association study in Estonian Biobank
GWAMA (v2.2.2): In-house developed software for genome-wide association meta-analysis (Mägi et al. 2010)
LDSC v1.0.1: Open-source software (Buli-Sullivan et al. 2015) for heritability and genetic correlation estimates (https://github.com/bulik/ldsc)
FUMA v1.3.6a: Online platform for functional annotation of GWAS results (Watanabe et al., 2017)
MAGMA v1.08: Open-source software (de Leeuw et al., 2015) was used to conduct gene and gene-set analysis
CTG-VL O.4-beta (Cuellar-Partida et al. 2019): Online platform for gene-set and tissue/cell-type enrichment analysis via DEPICT (Pers et al. 2015)
DEPICT v1: Gene-set and tissue/cell-type enrichment analysis software (Pers et al. 2015)
Msigdb v7.0: Gene sets were obtained from Msigdb v7.0 for "Curated gene sets" and "GO terms" as implemented in FUMAv1.3.6
ANNOVAR v2019Oct24: Open-source software (Wang et al. 2010) for variant annotation.
COLOC v3.2.1: Open-source R package (Giambartolomei et al. 2014) for colocalization analysis.
STEROID v0.1.1: In-house software to calculate PRS for all Estonian Biobank participants (https://genomics.ut.ee/en/tools/steroid)
PRSice 2 v2.3.3: Open-source tool (Eusden et al. 2017) for polygenic risk scoring
LDpred v1.0.11: Open-source tool (Vilhjalmsson et al. 2015) for polygenic risk scoring modelling
LDHub v1.9.3: Online tool (Zheng et al. 2017) for genetic correlation analysis
R 3.6.1. Open source software for statistical computing
ggplot2: Open-source R package for plotting data
survival: Open-source R package for survival analysis
pheatmap: Open-source R package for implementation of heatmaps

phenoscanner v1.0: Open-source R package which queries the PhenoScanner database of genotype-phenotype associations
GWAS Catalog e96_r2019-09-24: Open source database of genotype-phenotype associations
https://github.com/LappalainenLab/spiromics-covid19- eqtl/blob/master/eqtl/summary_phenoscanner_lookup.Rmd : this script was used to visualize phenoscanner results
Mouse Genome Database: We queried mouse mutant phenotypes utilizing Mouse Genome Database (MGI6.18, latest update from 05/04/2022) (http://www.informatics.jax.org/)

For manuscripts utilizing custom algorithms or software that are central to the research but not yet described in published literature, software must be made available to editors and reviewers. We strongly encourage code deposition in a community repository (e.g. GitHub). See the Nature Portfolio guidelines for submitting code & software for further information.

## Data

Policy information about availability of data

All manuscripts must include a data availability statement. This statement should provide the following information, where applicable:

- Accession codes, unique identifiers, or web links for publicly available datasets
- A description of any restrictions on data availability
- For clinical datasets or third party data, please ensure that the statement adheres to our policy

The full meta-analysis summary statistics generated in this study have been deposited in the GWASCatalog (https://www.ebi.ac.uk/gwas/) under accession code GCST90102470. The PRS summary statistics generated in this study have been deposited in the PGS Catalog (https://www.pgscatalog.org/) database under accession code PGS002288. The individual level data from Estonian Biobank are available under restricted access for containing sensitive information from healthcare registers, access can be obtained through the Estonian biobank upon submission of a research plan and signing a data transfer agreement. All data access to the Estonian Biobank must follow the informed consent regulations of the Estonian Committee on Bioethics and Human Research, which are clearly described in the Data Access section at https://genomics.ut.ee/en/content/estonian-biobank. A preliminary request for raw genetic and phenotype data must first be submitted via the email address releases@ut.ee. Icelandic and UKBB summary statistics can be accessed from http://www.decode.com/summarydata and FinnGen summary statistics can be downloaded after filling this form (https://elomake.helsinki.fi/lomakkeet/102575/lomake.html). We queried mouse mutant phenotypes utilizing Mouse Genome Database (MGI6.18, latest update from 05/04/2022) (http://www.informatics.jax.org/).

# Field-specific reporting

Please select the one below that is the best fit for your research. If you are not sure, read the appropriate sections before making your selection.

☒ Life sciences ☐ Behavioural & social sciences ☐ Ecological, evolutionary & environmental sciences

For a reference copy of the document with all sections, see nature.com/documents/nr-reporting-summary-flat.pdf

# Life sciences study design

All studies must disclose on these points even when the disclosure is negative.

| | |
|---|---|
| Sample size | The sample size consists of all individuals that remain after quality control of the data, including a total of 28,086 women with pelvic organ prolapse and and 546,321 controls of European ancestry. Detailed information on the samples used and inclusion/exclusion criteria are provided in the Supplementary Information. Sample size was obtained based on maximum sample size available in Estonian Biobank and also the identification and addition of all available publicly available summary statistics for this trait. |
| Data exclusions | See Supplementary Information. Individuals were excluded from the analysis if they failed quality control procedures, if their call-rate was < 95% or if their sex defined by heterozygosity of X chromosomes did not match their sex in the phenotype data. |
| Replication | All available datasets were included in the primary meta-analysis. We examined the concordance of effects within the novel loci (available in Supplementary Figure 2) and reported how our results replicate previous GWAS meta-analysis. The results of the functional follow-up were compared to the results of the previous GWAS in pelvic organ prolapse. Replication was not feasible since we included all publicly available datasets in the original analysis. |
| Randomization | In genome-wide association analysis case and control group were defined as presence or absence of N81 diagnosis, and genetic association analysis in Estonian Biobank were adjusted by age and first 10 principal components. Randomization was used to select controls in polygenic risk scores analysis in the discovery set (5,379 prevalent cases and 21,516 controls). The selection of controls in the discovery set was randomized, including 4 controls per case. Since controls were defined as women who did not develop pelvic organ prolapse during follow-up (which initiates in first linkage to Estonian Health Insurance Fund in 26-11-2002 and ends in latest linkage to diagnoses dating from October 30-12-2019). Cases were not otherwise matched to controls. A logistic regression was used to assess the discriminative ability towards case-control association of PRSs models. Analyses were adjusted by age, age squared, batch effects and first 10 principal components. In the other sets (validation set and subset of the PRS study) Cox proportional hazard models were used to estimate the Hazard Ratios (HR) corresponding to one standard deviation (SD) of the continuous PRS. Harrell's C-statistic was used to characterize the discriminative ability of each PRS. Cumulative incidence estimates were computed using Kaplan-Meier method. We used survival modeling, where age was used as a timescale to properly account for left-truncation in the data and right-censoring. |
| Blinding | The case control status of individuals was assigned through ICD-based coding. The analysts were not blinded to the status of the individuals because QC procedures require knowing case status and analysis from Icelandic, UKB and FinnGen were conducted using summary statistics |

with no individual-level data. Blinding was not perform during analysis from Icelandic, UKB and FinnGen data since it was required to properly evaluate heterogeneity of effects after performing the meta-analysis.

# Reporting for specific materials, systems and methods

We require information from authors about some types of materials, experimental systems and methods used in many studies. Here, indicate whether each material, system or method listed is relevant to your study. If you are not sure if a list item applies to your research, read the appropriate section before selecting a response.

## Materials & experimental systems

| n/a | Involved in the study |
|---|---|
| ☒ | ☐ Antibodies |
| ☒ | ☐ Eukaryotic cell lines |
| ☒ | ☐ Palaeontology and archaeology |
| ☒ | ☐ Animals and other organisms |
| ☐ | ☒ Human research participants |
| ☒ | ☐ Clinical data |
| ☒ | ☐ Dual use research of concern |

## Methods

| n/a | Involved in the study |
|---|---|
| ☒ | ☐ ChIP-seq |
| ☒ | ☐ Flow cytometry |
| ☒ | ☐ MRI-based neuroimaging |

# Human research participants

Policy information about studies involving human research participants

**Population characteristics**

Participants were of European Ancestry, restricted to females only according to the phenotype of interest (ICD-10 diagnose N81). Genetic association analysis in Estonian Biobank were adjusted by age and first 10 principal components. PRS analysis in the discovery set were adjusted by y age, age squared, batch effects and first 10 principal components. In PRS analysis in the validation set and subset, Cox proportional hazard models were used to estimate the Hazard Ratios (HR) corresponding to one standard deviation (SD) of the continuous PRS. Harrell's C-statistic was used to characterize the discriminative ability of each PRS. Cumulative incidence estimates were computed using Kaplan-Meier method. We used survival modeling, where age was used as a timescale to properly account for left-truncation in the data and right-censoring.

**Recruitment**

Participants were recruited from volunteer-based biobanks. We assessed and demonstrated the robustness of the POP association results by comparing the effect sizes between studies for the presented 30 lead variants (see Supplementary Figure 2). Biobanks are a key source for epidemiologic and genetic research, but bias may be introduced if those who accept the recruitment invitation differ systematically from those who do not. Oversampling or recruitment strategy changes may be necessary to increase participation among less-represented groups.

**Ethics oversight**

All participants have signed a broad informed consent form. This study was carried out under ethical approval 1.1-12/624 from the Estonian Committee on Bioethics and Human Research (Estonian Ministry of Social Affairs). For UKB, Icelandic and FinnGen we used publicly available summary level data for genetic association analyses, without additional ethics approval needed.
All necessary patient/participant consent has been obtained and the appropriate institutional forms have been archived.

Note that full information on the approval of the study protocol must also be provided in the manuscript.
